# Cholecystostomy does not prevent gallstone ileus: a case report

**DOI:** 10.4076/1757-1626-2-6790

**Published:** 2009-07-31

**Authors:** Pulathis Nilantha Siriwardana, Deepaka Weerasekara, Mohan De Silva

**Affiliations:** University Surgical Unit, Colombo South Teaching HospitalS De S Jayasinghe Road, Kalubowila, 10350Sri Lanka

## Abstract

**Introduction:**

Gallstone ileus following cholecystostomy has been reported once, in a patient with acute cholecystitis, where symptoms of small intestinal obstruction had developed one day after surgery. We report a case of gallstone ileus eight months following a cholecystostomy, which might deter the diagnosis. This is the only such reported case in medical literature according to our knowledge.

**Case presentation:**

A 54-year-old Sri Lankan female with a past history of a cholecystostomy presented with symptoms suggestive of small intestinal obstruction. Evidence of ileal obstruction with pneumobilia in the supine radiograph of the abdomen and cholecyto-duodenal fistula in the water soluble contrast study was suggestive of the diagnosis of gallstone ileus. An enterolithotomy was performed with no attempt of closure of the cholecysto-duodenal fistula.

**Conclusion:**

This case demonstrates the value of the supine radiograph of the abdomen and the barium follow-through in diagnosis. A cholecystogram, preferably preoperative, is the mainstay of prevention and identification of this clinical scenario.

## Introduction

Gallstone ileus following cholecystostomy has been reported only once, in which the patient developed symptoms of intestinal obstruction in the same admission [1]. The morbidity and mortality rate of gallstone ileus remain very high, partly because of misdiagnosis and delayed diagnosis [2]. A past history of a cholecystostomy may further deter the diagnosis, which may lead to disastrous consequences.

## Case presentation

A 54-year-old Sri Lankan female, with a past history suggestive of several episodes of acute cholecystitis, presented with central abdominal pain, distension and bilious vomiting for one day. She had a cholecystostomy during an attempt of an open cholecystectomy, due to obscured anatomy, eight months prior to presentation. The cholecystostomy tube was removed in ten days. However, a tube cholecystogram had not been performed.

On examination, she was moderately dehydrated, afebrile and anicteric. Her pulse rate was 90 per minute. She was haemodynamically stable. Abdominal examination revealed distension. However, it was soft and non tender. Bowel sounds were exaggerated. Digital examination revealed an empty rectum with no palpable mucosal lesions. A provisional diagnosis of adhesive intestinal obstruction was made and initial management was planned accordingly.

**Figure 1.**
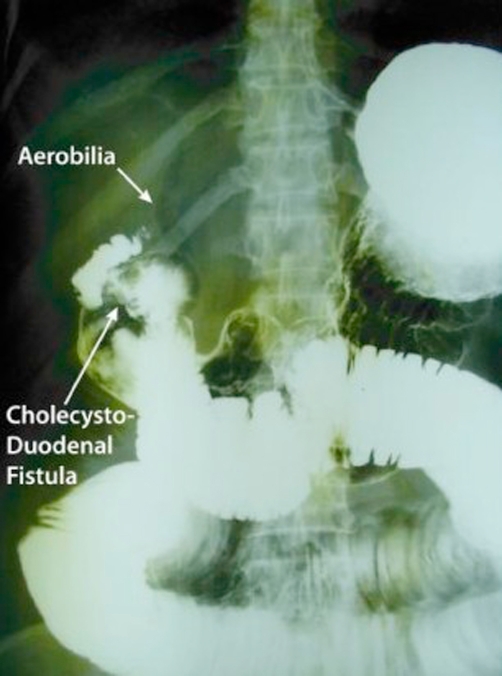
Roentgenogram of the barium follow through showing evidence of small bowel obstruction (dilated bowel loops) with aerobilia and the cholecysto-duodenal fistula.

Haematological investigations did not have any evidence of sepsis, anaemia or electrolyte imbalance. A supine abdominal x ray demonstrated features suggestive of upper small bowel obstruction and pneumobilia. A water soluble contrast study was performed to determine the cause of pneumobilia and intestinal obstruction, which revealed evidence of a cholecysto-duodenal fistula and mechanical obstruction of the small bowel. However, there was no radio opaque mass within the small bowel. Pneumobilia and evidence of the cholecysto-duodenal fistula in the presence of small intestinal obstruction was suggestive of gallstone ileus (Figure 1).

**Figure 2.**
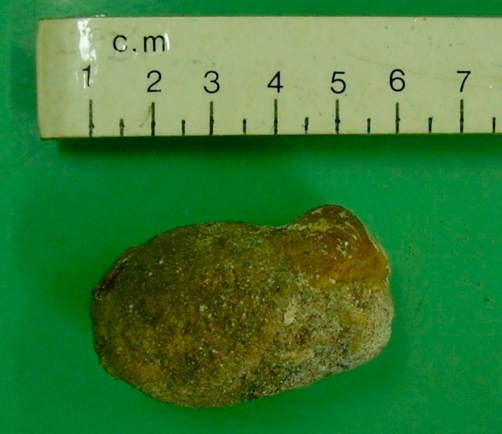
The gallstone following enterolithotomy.

Following initial resuscitation with intravenous fluid, pain relief, nasogastric suction and monitoring, an exploratory laparotomy was performed. Peritoneal survey revealed multiple adhesions not amounting to intestinal obstruction with obscured anatomy in the right hypochondrium. A 6 × 4 × 4 cm stone was found impacted in the terminal ileum about 15 cm proximal to the ileo-caecal valve (Figure 2). Enterolithotomy and small bowel decompression was performed. The closure of the cholecysto-duodenal fistula was not attempted. Recovery was uneventful and the patient is well at 18 months of follow up.

## Discussion

Cholecystostomy may be a life saving procedure for high-risk patients with acute cholecystitis or peri-cholecystic abscess. Occasionally it is indicated for cholelithiasis when cholecystectomy proves unduly difficult and potentially hazardous even in otherwise healthy patients [3,4]. A postoperative tube cholecystogram is indicated prior to removing the cholecystostomy tube. However, this had not been performed. A tube cholecystostogram, performed to identify overlooked calculi, would have also revealed the cholecysto-duodenal fistula.

Gallstone ileus is mechanical intestinal obstruction due to impaction of one or more large gallstones. It is a potentially serious complication of cholelithiasis [5], which occurs in 0.5% of cases [6]. Biliary-enteric fistula is the major pathologic mechanism of gallstone ileus. Small diameter and less active peristalsis make the terminal ileum and the ileo-caecal valve the commonest sites of impaction and the less common sites are jejunum, Ligment of Treitz and the stomach while the duodenum and the colon are rare locations of impaction [6].

Diagnosis of gallstone ileus is difficult and often delayed with 50% of cases detected only at laparotomy [6]. The classic Rigler’s triad of radiography includes mechanical bowel obstruction, pneumobilia, and an ectopic gallstone within bowel lumen, which is best demonstrated by abdominal CT (78%) [7]. This patient’s plain film of abdomen revealed dilated loops of small intestine and pneumobilia. However, it did not show an ectopic gallstone. This is likely, because only 10% of biliary calculi are sufficiently calcified to be visualised radiographically. As reported by Balthazar EJ et al, demonstration of a fistulous tract adjacent to the first portion of the duodenum associated with jejunal dilatation and barium dilution was highly reliable of gallstone ileus [8] (Figure 1).

The mortality rate is in the order of 7.5%-15%, largely due to delayed diagnosis [6,9]. Hence treatment requires urgent surgery. This patient had an enterolithotomy with no attempt of a cholecystectomy and fistula closure. Surgical strategies are ‘enterolithotomy alone’ and ‘enterolithotomy combined with cholecystectomy and fistula closure’. Tan et al compared these two surgical strategies and concluded that both procedures are safe with no mortality, but supports ‘enterolithotomy alone’ as the better option [10].

## Conclusion

This article contributes to medical literature as the second reported case according to our knowledge, where gallstone ileus has presented in a patient following cholecystostomy. In contrast to the previous case, where symptoms appeared in the same setting one day after surgery, this patient presented eight months after cholecystostomy, which is unique. This report illustrates the importance of a postoperative cholecystogram, preferably per-operative. The cholecystogram would have revealed the cholecysto-duodenal fistula. Hence, we recommend exploration for a cholecysto-duodenal fistula by an on table cholecystogram or by examining the interior of the gallbladder during cholecystostomy. The presence of a fistula is an indication for careful examination of the intestine for a gallstone. In the absence of CT facilities, the radiograph and gastrografin study are diagnostic of gallstone ileus.

## Abbreviation

CT: Computed tomography
